# Intervention and Improved Well-Being of Basic Science Researchers During the COVID 19 Era: A Case Study

**DOI:** 10.3389/fpsyg.2020.574712

**Published:** 2020-11-09

**Authors:** Santosh Kumar, Sunitha Kodidela, Asit Kumar, Kelli Gerth, Kaining Zhi

**Affiliations:** ^1^Department of Pharmaceutical Sciences, College of Pharmacy, University of Tennessee Health Science Center, Memphis, TN, United States; ^2^The Plough Center of Drug Delivery Solutions, University of Tennessee Health Science Center, Memphis, TN, United States

**Keywords:** COVID-19, productivity, perceived stress score, laboratory research, well-being

## Abstract

The coronavirus disease-19 (COVID-19) pandemic has affected individuals of all categories, irrespective of their geographical locations, professions, gender, or race. As a result of full or partial lock-down and stay-at-home orders, the well-being and productivity of individuals were severely affected. Since basic science research requires laboratory experiments, the work-from-home strategy hurt their productivity. In addition, the combination of decreased productivity and staying at home is likely to compromise their well-being by causing stress and anxiety. In this case study, a strategy was developed to engage researchers through listening and learning, motivation, and empowerment, using regular virtual sessions. Through these virtual sessions, research work was prioritized and coordinated, from idea conception to writing research papers and grant proposals. Perceived stress scores (PSS) and COVID-19-related stress (COVID-SS) scores were measured to evaluate general and COVID-19-induced stress, respectively, every month from March to July 2020 during the COVID-19 era. The result showed a significant improvement in both the PSS and the COVID-SS scores of the intervention group compared to the control group. In addition, while there was no/minimal change in PSS and COVID-SS scores from March to subsequent months until July for the control group, the intervention groups showed significant and consistent improvement in both scores in the intervention group. Overall, the intervention strategy showed improved well-being for basic science researchers, which was also consistent with their improved productivity during the COVID-19 era.

## Introduction

The coronavirus disease-19 (COVID-19) pandemic is an ongoing world crisis. This pandemic has taken a toll on human health and has also placed a huge burden on economies, societies, and families across the globe (Carter et al., 2020; Cutler, 2020; Donthu and Gustafsson, 2020; Hua et al., 2020; Jenson, 2020; Mclaren et al., 2020; Ornell et al., 2020; Power, 2020; Satiani et al., 2020). This COVID-19 crisis is further deepened because the future of countries, societies, and individuals is uncertain and unpredictable in the months and perhaps years to come. A recent special issue on COVID-19 by “Taloy and Francis” describes the impact of this pandemic on, “Emerging markets finance and trade,” which ultimately causes stress in world economies and societies (Taylor and Francis, 2020). In addition to the impact on world economies, world trade has experienced a massive contraction as a result of a drastic reduction in trade connectivity and commercial activities among countries during COVID-19 outbreak (Vidya and Prabheesh, 2020). The trade forecast among the major trading countries further Shows a decline until December 2020. However, it is worth mentioning that amid the COVID-19 pandemic, there is a significant improvement in air quality, though temporarily, and a positive macroeconomic response has been seen in some countries such as China and India during the COVID-19 outbreak (Ming et al., 2020). The impact on global economies and loss of millions of jobs have been one of the major causes of stress and anxiety among global populations.

The impact of the COVID-19 pandemic on human health, which caused ∼38 million infections and >1 million deaths world-wide as of October 15, 2020, far exceeds the impact of previous epidemics or pandemics in recent history (Coronavirus-Resources-Center, 2020). Although over 90% of people recovered from the infection/disease, many individuals suffered from multiple organ damage (lungs, kidney, liver, heart, etc.) (Renu et al., 2020; Spuntarelli et al., 2020). Further, a large number of recovered populations from COVID-19 also suffered from mental and psychological diseases/conditions such as stress, anxiety, and depression (Salari et al., 2020; Xiong et al., 2020). Studies have shown that ∼50% of individuals who recovered from COVID-19 are diagnosed with depression, and ∼40% are diagnosed with anxiety and stress (Rogers et al., 2020). Individuals associated with COVID-19 patients, and others, especially those who have lost their jobs and are experiencing financial crises, also show symptoms of depression, anxiety, and stress (Dubey et al., 2020; Titov et al., 2020).

This is the first time in modern history that almost all countries, either fully or partially, enter into a lock-down phase and enforce stay-at-home orders (Asensio et al., 2020). One of the major health concerns, as a result of lock-down and stay-at-home orders, is the mental health of individuals who stay at or work from home (Killgore et al., 2020). Stress and anxiety are usual reactions to any unpredictable pandemic situation. As a result of stress due to the COVID-19 pandemic, the general population, particularly health care professionals and college students, experienced changes in concentration, anxiety, irritability, and eventually reduced productivity (Tangen et al., 1981; Kecojevic et al., 2020; Ozamiz-Etxebarria et al., 2020; Stanton et al., 2020; Wu et al., 2020). These studies suggest a need to develop mitigation and psychological intervention strategies that can improve the mental health of the general population during the COVID-19 era, especially in vulnerable groups such as health professionals and college students. To the best of our knowledge, there is no study conducted among basic science researchers to examine the impact of the COVID-19 epidemic on psychological health and stress or the relationship of these factors to productivity. Therefore, we conducted a case study on intervention and well-being of basic science researchers at the University of Tennessee Health Science Center (UTHSC).

Research laboratories at UTHSC were closed for all non-emergency work in March, and the researchers were asked to work from home (UTHSC, 2020). Although manuscripts and grant writing could be done from home, it is very difficult to stay productive if experiments in the basic science laboratory are completely stalled. Basic science experiments take 1–2 weeks to wrap up and equally the same time to restart. Thus, until the research laboratories partially opened in the first week of June, employees had lost 3 months of complete followed by 2 months (June–July) of partial basic science research. In addition to reduced productivity, the work-from-home plan for researchers who normally work in a laboratory setting can increase stress and anxiety. Compounded by COVID-19-related stress, this has the potential to further reduce productivity. Moreover, due to uncertainty surrounding lab reopening dates, researchers were also uncertain about their career progression. All these factors may contribute to a lack of concentration, irritability, insomnia, and reduced productivity among scholars.

The objective of the present study is to design an interventional strategy to mitigate stress and maintain well-being and productivity for basic science researchers during the work-from-home order in the COVID-19 era. The mitigation strategy is to plan and implement necessary experiments during the pre-lock-down period, followed by engaging in idea development, data analysis, and manuscript writing, as well as engaging in listening and empowering sessions via virtual lab meetings during and after the lock-down periods. The hypothesis is that the interventional strategy will significantly reduce stress and improve the well-being of basic science researchers while maintaining their productivity. To assess the well-being of subjects, Perceived Stress Score (PSS) and COVID-19-related stress scores (COVID-SS) were measured. Generally, the stress levels of health care professionals and college students are measured using the PSS method (Du et al., 2020; Georgiou et al., 2020; Guo et al., 2020; Meira et al., 2020; Zarghami et al., 2020), which is the most widely used method to monitor perceived stress (New Hampshire Department of Administrative Services, 2020). However, to measure the stress, anxiety, and overall well-being of individuals specifically induced by COVID-19-related changes in lifestyle and altered productivity, the PSS method may not be sufficient. Therefore, we used the COVID-19 stress related score (COVID-SS) to assess the fear, learning, and growth in knowledge of individuals during the pandemic (Epilepsy Society, 2020). The current study results suggest an improved well-being of the intervention group compared to the control group, which is also consistent with the reduced stress and improved productivity of the intervention group.

## Methods

### Preparation Before the Crisis for Intervention Group

When the WHO declared COVID-19 a Public Health Emergency of International Concern on 30 January 2020 (Patel et al., 2020), a strategic plan for researchers in our group was put-together. The strategic plan included: (1) postponing manuscript writing and other paper work and performing wet-lab experiments to obtain data until the lab was closed in the second week of March, (2) data analysis and manuscript writing during the work-from-home orders from mid-March to May 31 and until July 31 during partial lab-closure, (3) conceiving new ideas and writing manuscripts for review papers, as well as writing grant proposals for the same periods. To make the researchers accountable for their productivity, a 2 h virtual lab meeting every Monday and one-on-one virtual meetings as needed were implemented. The demographics of the intervention group was 4 men and 5 women that included 3 students, 2 post-doctorate fellows, 3 research staffs, and 1 faculty. The study population was generally healthy and their age ranged approximately from 22 to 50 years. Since the intervention requires a certain supervisory relationship among all participants, it is not feasible to increase group size. Inviting researchers from other research groups may result in a conflict of interest among principle investigators since most research groups are independent. Hence, we could include only nine people in the intervention group.

### Implementation During the Crisis

A modified anonymous strategy was used as an intervention. Almost half of each lab meeting until May 31 was spent in listening to everyone’s concerns, celebrating any good news, and COVID-19-related facts from reliable sources. The frequency of these discussions was reduced when the laboratory was partially opened from June 1 to July 31. In general, the strategy was to learn from each other and empower each other. During the laboratory meetings, some engaging games were also played to overcome stress. The empowering sessions were developed based on vast knowledge, emotional intelligence, and the experience of our diverse group, as well as available literatures (World Health Organization, 2004; Shultz et al., 2016; Hendriks et al., 2017; Seyedin et al., 2019; Jiménez et al., 2020; Schlesselman et al., 2020). We compiled the following discussion topics to empower each other.

(1) COVID-DIFFERENTIATOR (COVID-DIFF): Similar to any crisis, COVID would differentiate people into three categories: (1) Individuals who were negatively impacted (with no mistake of theirs), (2) individuals who stayed the course and were able to handle well, and (3) individuals who found new opportunities and improved performance. In general, most people, including our study participants, belong to categories 1 and 2. Our goal was to empower them with the below mentioned strategies, which could help them to move to category 3.
(2) Faith/dreams vs. Panic/fear: The intervention group discussed the pros and cons of having faith/dreams vs. feeling panic/fear, with numerous examples. These empowered each other to have faith and dreams.
(3) Facts/reality vs. Opinion/hype: The intervention group was advised to follow facts and reality and educate others with these rather than uncorroborated opinions and hype.
(4) Safety vs. Carelessness: The intervention group was educated to exercise safety and caution by following the COVID-19 policies and guidelines of national and local organizations.
(5) Managing the crisis vs. Getting under the crisis: The intervention group discussed various aspects of the crisis and how one can manage the crisis, rather than getting under the crisis, in a way that negatively impacts us.
(6) Thriving vs. Surviving: Finally, the intervention group discussed how to thrive during the crisis and not just survive. As Stanford economist Paul Romer once stated, “a crisis is a terrible thing to waste” (Chisholm-Burns, 2010). The intervention group as a whole decided, “we will not let the crisis go to waste.” The group discussed various ways to improve productivity and manage stress during the crisis. For example, ways to improve grit and mental toughness by acquiring positive attitudes and self-discipline were discussed. Besides, performing physical and mental activities, such as walking/running/exercising, yoga, and meditation were promoted in group discussion.

In addition to the above empowering sessions, the intervention group also discussed the following advantages of working from home.

(1) Freedom: freedom to work with a chosen time, place, and uniform.
(2) Family together: opportunity to spend quality and quantity time with families.
(3) Time to think creatively: compared to lab and office environments, work-from-home may give a change in environment, more time, and quietude to think creatively.
(4) Yoga and Meditation: a home environment may empower people to do yoga and meditation to maintain physical and mental health.
(5) Opportunity to take care of the backlog, start new writing projects, and contribute to society: working from home may give more time for data analysis, writing manuscripts, and initiating new projects for review papers and/or grant proposals. It can also motivate and empower society, which is going through a difficult time, through messages via reliable sources.

Finally, as a group and as individuals, the intervention group did reflection exercises on the following things. (1) How have I contributed positivity or negativity to others? (2) Does someone feel better after an interaction with me vs. how they felt before? (3) Did shared interest rise above self-interest? (4) Did I listen more – or talk more? (5) How many times today did I complain about someone or something? (6) How many times did I simply say thank you? (7) What did I learn this week, especially that challenged my thought processes? (8) What did I do this week, especially that is unique and out-of-norms?

#### Control Group

A control group of UTHSC basic science researchers, which did not go through the intervention as described above, is included in this study. The control group consists of 6 students, 3 post-doctorate fellows, and 1 research scientist (5 men and 5 women). The participants were generally healthy and their age ranged approximately from 25 to 40 years. The control group of basic science researchers also went through similar challenges at UTHSC due to complete lab-closure from mid-March to May 31 and partial lab-closure from June 1 to July 31.

## Outcome Measures

Two outcomes were measured during the 5-month period. The PSS and COVID-SS outcomes were measured by using their respective surveys upon an Institutional Review Board (IRB) approval from the University of Tennessee Health Science Center.

### Perceived Stress Score (PSS)

The PSS of nine participants from the intervention group and ten participants from the control group for the months of March–July were measured, upon their consent to do a volunteer survey. PSS is the most-widely used method to measure stress levels in occupational health, especially among professional students in health science. This method was essentially used as described (New Hampshire Department of Administrative Services, 2020). In brief, PSS was measured by self-scoring the following questions. Scoring was performed (between 0 and 4; 0 being never and 4 being very often), followed by reversing the scores of questions 4, 5, 7, and 8, and then adding all the scores. Scores with 0–13, 14–26, and 27–40 are defined as low, moderate, and high stress, respectively. The group PSS scores were then analyzed longitudinally for the months of April–July, using March as control month, as the intervention began in April. COVID-SS scores for the intervention group were also compared and analyzed from the control group for each month.

### COVID-19-Related Stress Scores (COVID-SS)

The COVID-SS of nine intervention participants and ten control participants for the months of March–July were also measured upon their consent to do a volunteer survey. COVID-SS is a new method that used to assess the stress level of participants during the COVID-19 era using their behaviors and actions in three zones (fear, knowledge, and growth). This method was essentially used as described previously (Manch, 2020). In brief, the questions/statements, as presented in Table 1, were used to self-assess the three zones: fear, knowledge, and a growth mindset. Every correct statement for each zone carries one point. The total points for each zone represent the mindsets and attitudes of participants in terms of COVID-19-related fear, knowledge, and growth. The information obtained from these zones can then be correlated with COVID-19-induced stress and overall well-being of participants. The group COVID-SS scores were then analyzed longitudinally for the months of April-July, using March as the control month, as the intervention began in April. The COVID-SS scores for the intervention group were also compared and analyzed from the control group for each month.

**TABLE 1:** Statements used to score COVID-SS for each zone. Each correct statement carries 1 point.

Fear zone (total 5 points)	Knowledge zone (total 7 points)	Growth zone (total 8 points)
I grab food, medications, and toilet paper that I don’t need	I start to give up what I can’t control	I think of others and know how to help them
I spread emotions related to fear and anger	I stop consuming what hurts me, from food to news	I make my talents available to those who need them
I complain frequently	I identify my emotions	I live in the present and focus on the future
I forward all messages I receive about COVID-19	I am aware about the situations and know how to act	I am empathetic to myself and to others
I get mad easily	I evaluate information before spreading false	I thank and appreciate others
	I recognize that we all are trying to do our best	I keep a happy emotional state and give hope
		I look for a way to adapt to changes
		I practice quietude, patience, relationships, and creativity

### Research Productivity

Our mitigation and empowering strategies were likely to improve the research productivity. It was measured only in our intervention study group in terms of conceiving ideas, data analysis, manuscript writing and submission, manuscript acceptance, and publication, as well as grant submission.

### Statistical Analysis

Mean ± SEM was calculated and compared to the control group. Student’s *T*-test was applied to compare the scores between the intervention and control groups, as well as between the control month (April) and individual intervention months (April–July) for both control and intervention groups. All the statistical calculations were performed using GraphPad Prism 7. *p* < 0.05 was considered statistically significant.

## Results

### Perceived Stress Score (PSS)

**FIGURE 1:**
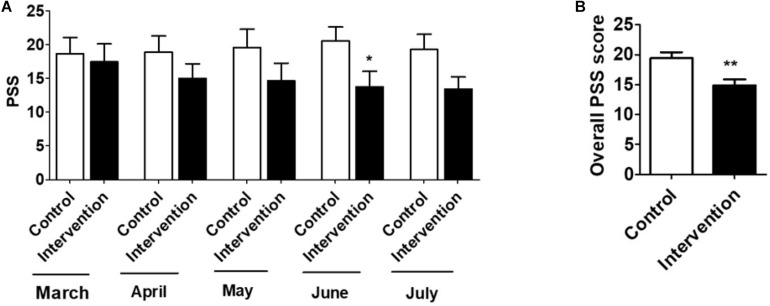
**(A)** Mean ± *SD* of Perceived Stress Score (PSS) of control (*n* = 10) and Intervention (*n* = 9) groups for March to July. **(B)** Mean ± *SD* of overall PSS of control (*n* = 50, 10 subjects for 5 months) and intervention (*n* = 45, 9 subjects for 5 months) groups. *T*-test was applied to compare the scores between intervention and control groups. *p* < 0.05, *p* < 0.01 are represented as “*” and “**”, respectively when compared the scores between intervention and control groups. “#” represents *p* < 0.05 when compared between intervention groups (March vs. other months). “$” represents *p* < 0.05 when compared between control groups (March vs. other months).

An intervention group of nine participants and a control group of ten participants volunteered to take the perceived stress test, as described in the outcomes measure section. The Mean ± *SD* of the PSS were evaluated, and the relative scores of the intervention group vs. control group were analyzed. Comparison and analysis were also performed in a longitudinal manner, in which March was a control month when the intervention began (Figure 1). Overall, results showed a relatively high PSS (17.4 ± 2.7) for the intervention group in March, which consistently decreased in the subsequent months, with a statistically significant decrease in June (13.8 ± 2.3) (Figure 1A). However, the PSS scores did not significantly change in the control group from the months March to July. Importantly, there was a statistically significant decrease in the overall PSS scores (March-July combined) of the intervention group compared to the control group (14.7 ± 0.8 vs.19.3 ± 0.3) (Figure 1B). In general, the intervention group showed an increased stress level (moderate stress) in March, which was subsequently decreased to low stress in the subsequent months. However, the stress level in the control group remained moderate throughout these 5 months. Since the PSS method is used to measure general stress levels, in the following section we used COVID-19-related stress scores in our participants and determined whether intervention group had a significantly different stress level.

### COVID-19-Related Stress Scores (COVID-SS)

**FIGURE 2:**
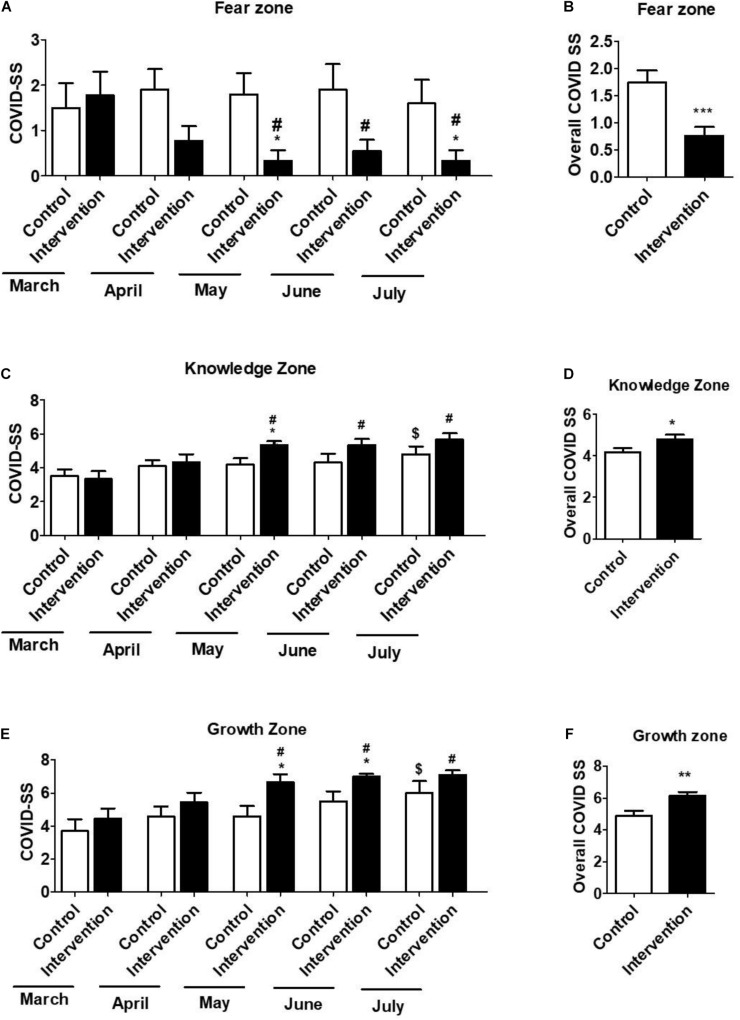
The COVID-related stress score (COVID-SS) in the fear zone **(A,B)**, knowledge zone **(C,D)**, and growth zone **(E,F)** for March, April, May, June, and July were collected and compared between control (*n* = 10) and intervention (*n* = 9) groups. The scores of intervention and control groups in March were also compared to their respective scores of intervention and control groups in other months in each zone. The data in **(B,D,F)** represent Mean ± *SD* of overall COVID-SS scores of control (*n* = 50, 10 subjects for 5 months) and intervention (*n* = 45, 9 subjects for 5 months) groups. *T*-test was applied to compare the scores between months. *p* < 0.05, *p* < 0.01 are represented as “*” and “**” or “***”, respectively when compared the scores between intervention and control groups. “#” represents *p* < 0.05 when compared between intervention groups (March vs. other months). “$” represents *p* < 0.05 when compared between control groups (March vs. another month).

COVID-SS measures three different components (fear, knowledge, and growth zones) as described in the outcomes measure section. This method was used specifically to measure COVID-19-related stress and anxiety. COVID-SS examines whether participants can change their behavior and actions as a result of training and move from the fear zone to the knowledge zone, and ultimately the growth zone, across the 5 months. Nine participants from the intervention group and ten participants from the control group took the COVID-19-related stress test survey. The Mean ± *SD* of COVID-SS was evaluated for each zone during the months of March–July. The results from nine intervention participants showed a relatively high COVID-SS for the fear zone (1.78 ± 0.52) in March, which subsequently decreased in April, with a statistically significant decrease in May (0.33 ± 0.23), June (0.55 ± 0.24), and July (0.33 ± 0.23) (Figure 2A). On the other hand, the COVID-SS for the knowledge zone steadily increased from March to July, with a statistically significant increase in May (5.23 ± 0.23), June (5.33 ± 0.37), and July (5.66 ± 0.37) compared to March (3.33 ± 0.47) (Figure 2C). Similarly, the COVID-SS for the growth zone also steadily increased from March to July, with a statistically significant increase in May (6.67 ± 0.41), June (7.01 ± 0.16), and July (7.10 ± 0.26) compared to March (4.44 ± 0.62) (Figure 2E). On the other hand, compared to march, COVID-SS scores of the control group in the fear zone did not statistically change in the subsequent months (Figure 2A). However, compared to March, COVID-SS scores in July significantly increased in both knowledge (4.80 ± 0.49 vs. 1.27 ± 0.42) (Figure 2C) and growth (6.01 ± 0.75 vs. 2.26 ± 0.75) zones (Figure 2E), perhaps due to partial opening of the lab. However, this increase in the knowledge and growth zones for the control group was relatively lower than that of the respective increase in the intervention group.

More importantly, COVID-SS scores of the intervention group in the fear zone were significantly lower than the control group in May (0.33 ± 0.22 vs. 1.47 ± 0.49) and July (0.33 ± 0.33 vs. 1.65 ± 0.55) (Figure 2A). On the other hand, COVID-SS scores of the intervention group in knowledge zone were significantly higher than the control group in May (5.33 ± 0.23 vs. 1.13 ± 0.38) (Figure 2C). Similarly, COVID-SS scores of the intervention group in growth zone were also significantly higher than the control group in May (6.67 ± 0.47 vs. 1.95 ± 0.65) and June (7.01 ± 0.16 vs. 1.90 ± 0.63) months (Figure 2E). We also analyzed the overall COVID-SS scores for each zone for the months of March-July for both intervention and control groups. The overall COVID-SS scores of the intervention group in the fear zone were significantly lower than the control group (0.75 ± 0.26 vs. 1.74 ± 0.08) (Figure 2B). On the other hand, the overall COVID-SS scores of the intervention group in the knowledge zone were significantly higher than the control group (4.80 ± 0.43 vs. 4.18 ± 0.21) (Figure 2D). Similarly, overall COVID-SS scores of the intervention group in the growth zone were also significantly higher than the control group (6.13 ± 0.51 vs. 4.88 ± 0.40) (Figure 2F). Taken together, these findings suggest that intervention strategy to deal with COVID-related stress and anxiety significantly and consistently decreased the fear and increased the knowledge and subsequent growth in their knowledge.

### Productivity During the COVID-19 Era

It is widely known that reduced stress enhances productivity, and increased productivity feeds into low stress and improved well-being (Anderzén and Arnetz, 2005; Heylighen and Vidal, 2008). Stress and productivity work as a loop that feed into each other. Therefore, the research productivity was measured in terms of conceiving new ideas for a review paper, data analysis for the original paper, and manuscript writing and their publication in peer-reviewed journals. Since the evidence for only published papers can be provided, the productivity metrics for only published papers are presented in Table 2. Briefly, the data from a project (Table 2) was analyzed, which was later written and published. Two other manuscripts for original articles were also revised and published during the same time-period. In addition to original articles, 7 review papers and 1 editorial were published between March and the first week of September (Table 2). Two of these review papers are from the field of COVID-19 for which we conceived the idea of the paper during the COVID-19 era.

**TABLE 2:** Number of manuscripts written and published during the months of March–July.

PMID/DOI/In press	Type of paper	Idea	Data analysis	Manuscript submission	Revision submission	Published
PMID 32481515	Original article		X	X	X	X
PMID 32443728	Original article				X	X
PMID: 32433651	Original article				X	X
PMID 32696265	Editorial			X		X
PMID 32357553	Review	X		X	X	X
EIDDJ-100021	Review	X		X	X	X
PMID: 32722629	Review			X	X	X
PMID: 32823684	Review	X		X	X	X
doi: 10.1080/23808993.2020.1812382	Review				X	X
PMID: 32842791	Review				X	X
PMID: 32932786	Review			X	X	X

*“X” Represent completed task in that particular section.*

For the past 5 years, the average peer-reviewed publication rate for the group is 8 per year. Thus, publishing 11 papers in 6 months can be considered higher than the previous productivity for this research group. It has been widely accepted that obtaining data is the most time-consuming step and requires significant manpower. However, in the absence of experiments, optimal priorities and time management were implemented to maximize productivity with an overall exceptional result. The productivity is also considered unique, since two review articles were published on the much-needed field of COVID-19.

In addition to scientific papers, two opinion columns on COVID-19 were published in the Memphis-based “Commercial Appeal,” the “USA Today” network, on April 13 (Kumar, 2020b) and on June 11 (Kumar, 2020a). The former opinion column was on, “University of Tennessee Health Sciences Center making strides in treating COVID-19,” in which, a scientific opinion on repurposing antiviral drugs was provided. The latter one was on, “Challenges with COVID-19 could bring transformational change, improve human health,” in which, a scientific opinion on how COVID-19 could help improve general immunity and reduce the prevalence of chronic diseases was provided.

## Discussion

The present study was designed to mitigate general as well as COVID-19-induced stress in basic science researchers, which subsequently helps to improve the overall well-being and productivity in the intervention group. We used both PSS and COVID-SS methods to measure their stress levels and correlated the improved well-being of the intervention group with their productivity. Overall, findings strongly suggest that the mitigation strategy resulted in reduced stress levels and increased research productivity among basic science researchers during the COVID-19 pandemic. However, the data from the control group suggests that the current COVID-19 pandemic has a significant impact on the mental health of basic science researchers, which is consistent with the impact on mental health in the general population, especially in health care professionals and college students (Tangen et al., 1981; Kecojevic et al., 2020; Ozamiz-Etxebarria et al., 2020; Stanton et al., 2020; Wu et al., 2020).

Overall, the intervention group showed reduced general stress compared to the control group. Our outcome is different from the outcomes derived from the perceived stress and anxiety in the general population, in which this pandemic increased anxiety levels. In one study, high PSS scores among the general population were observed in women, persons under age 30, students, and those who believed themselves to be at a greater risk of contracting the illness (Limcaoco et al., 2020). Additionally, participants’ perception of susceptibility to COVID-19 was likely affected by several factors. Participants were not elderly or in other high-risk groups. Further, a certain level of scientific literacy (undergraduate and above) may have equipped the researchers to practice appropriate COVID-related health measures and mitigate COVID-related fear. Moreover, upon comparing with the control group, which were of similar demographics, ages, and education levels, it can be said that the strategy to deal with stress during the COVID-19 era has helped to manage stress levels of the researchers.

Intervention study based on the psychological health status of researchers as backline workers could provide a potential statewide measure that could be used by other researchers or even frontline workers to cope with stress during the pandemic outbreak. However, stress assessment and outcome measurements used in our study will be more appropriate for stress management and wellbeing of the mental state among researchers. Inconsistent with our findings, the frontline health care professionals, who were working in proximity to patients admitted in the ICU with severe lung infections, experienced mental health problems with substantial psychological distress (Greenberg et al., 2020). A descriptive study that was performed on health care professionals during COVID-19 revealed a relatively moderate level of perceived stress (PSS mean = 15.71 ± 4.02) on PSS-10, along with 38% identified as depressed and 24% as suffering from anxiety. Health care professionals who experience higher perceived stress than others likely worked at intensive care units (ICUs) (Ma et al., 2020). Findings of a meta-analysis indicated a high psychological impact, not only on healthcare workers (HCW) and patients, but also in the general population (Luo et al., 2020; Pappa et al., 2020). The psychological distress was mediated by anxiety and depression. However, the existence of other variables could be wrongly predicted as stress associated with COVID−19.

In a cross-sectional study conducted on frontline nurses (*n* = 325), 123 nurses were found to have a dysfunctional level of anxiety that involves fear, behavior, and psychological distress (Labrague and De Los Santos, 2020; Lee, 2020). Studies conducted on the psychological impact of COVID-19 on frontline nurses have found an overall high prevalence of anxiety ranged between 18 and 92.3% (Alwani et al., 2020; Luo et al., 2020) that could be averted by providing better organizational and social support, in addition to the implementation of safety measures at the workplace and quality personal protective equipment (PPE) (Labrague and De Los Santos, 2020). Overwhelming workload and lack of sleep may also contribute to the mental burden of frontline workers (Lai et al., 2020) that could be considered during the assessment of their stress levels. In general, healthy people were found to be less affected by COVID-19 related stress compared to those with anxiety-related or mood disorders in the population-based study conducted in the US and Canada (Asmundson et al., 2020). A cross-sectional survey based on modified PSS-10 conducted on 406 individuals comprising professors, students, and health professionals, aimed to assess the prevalence and variables related to perceived stress associated with COVID-19 (Pedrozo-Pupo et al., 2020). In total, 15% of the participants scored for high perceived stress associated with COVID-19. However, the prevalence of high perceived stress was relatively lower than previous studies performed during other epidemics, such as equine influenza (Pedrozo-Pupo et al., 2020). However, psychological responses to epidemics and outbreak management relate to several variables, such as misinformation or information overload and education, although findings regarding education can be inconsistent across different countries. For instance, less educated young people were found more vulnerable to high psychological distress during the outbreak of equine influenza in Australia (Taylor et al., 2008), whereas an opposite trend is seen in China (Qiu et al., 2020). Since PSS data is a test for well-being in general conditions, and it may be biased for stress induced by COVID-19, another method that measured COVID-SS was used.

The present study findings suggest that the intervention strategy to deal with COVID-related stress and anxiety significantly and consistently decreased the fear and increased the knowledge and subsequent growth in their knowledge. This is a new test that used for the first time to evaluate fear, knowledge, and growth mindsets in researchers during the COVID-19 era. Thus, it is not feasible to directly compare these outcomes with others in the literature that used different tests. This outcome measurement was used specifically in the context as an innovative strategy to help manage stress and increase productivity among researchers. Recent studies evaluated mental health associated with COVID-19-mediated stress and anxiety in the general population (Liu et al., 2020; Shammi et al., 2020), as well as in health workers who were involved in the treatment of COVID-19 patients (Bohlken et al., 2020; Yin et al., 2020). The outcomes from all those studies showed a significant decrease in their mental health as measured by the prevalence and predictors of post-traumatic stress symptoms (PTSS) and other methods. The participants in those studies experienced high stress and anxiety, lack of sleep, and uncertainty in their future. Thus, unlike other reports, outcomes from the current study with significant improvement in mental health suggest that the strategy to manage the stress of researchers appears to be effective. However, it is important to note that participants were at low risk of becoming unemployed and were not otherwise economically affected by the pandemic. Further, no participants in this study were directly affected by the illness; neither participants nor participants’ family members contracted the illness or suffered negative physical health outcomes related to the pandemic, and participants were not in high-risk groups for contracting the disease. In addition, most participants were not directly exposed to sick patients, in contrast with frontline workers. However, it can also be noted that the strategy helped to manage the well-being of the intervention group compared to the control group, which belonged to the same demography, age group, education level, and overall environment.

The United States has been experiencing a surge increase of anxiety prescription drugs in recent decades (Ross et al., 2019). The COVID-19 pandemic may exaggerate stress and anxiety issues in the US. The rationale of the current intervention study aims to provide a proof-of-principle to use anonymous based interventions as an alternative. Both PSS and COVID-SS scores are markers of stress management. It has been reported that group anonymous if performed properly, has the potential to turn negative stress into positive motivations (Murphy Lawrence and Hurrell Joseph, 1987). Anonymous is a widely used therapy method for treatment in alcohol, smoking, and narcotic drug abuse (Moos and Moos, 2006). In this study, the intervention emphasizes positive feedback, encouragement, and mental support to eliminate the fear, stress, and uncertainty due to COVID-19. Improvement in both PSS and COVID-SS scores from the intervention group, as well as relatively improved scores compared to the control group, proved the general improvement in stress conditions.

It is well-known that increased stress can significantly impair the productivity, and our mitigation strategy has improved the mental health and resulted in improved research productivity during the pandemic. Health and productivity management (HPM) was initially introduced back in the 1990s (Goetzel and Ozminkowski, 2000). The main goal of HPM was to train employees with the capability to handle crises and challenges. Stress management was also introduced at the beginning of the 21st century to promote productivity (Razavi et al., 2012). The COVID-19 pandemic is a challenge for both business and the community. Hence, training researchers to do more with few resources will benefit them in both the short-term and long-term. In the short-term, researchers are engaged in expanding their productivity portfolio by substituting wet-lab research to paper/computer-based research. The paper/computer research conducted during this period, including peer-reviewed articles and review paper writing and white/technical paper publications, are also valuable for their career. More importantly, these works, especially the process of literature research, may provide hints for future wet-lab experiments. It has been widely accepted by scientists that stepping away from the wet-lab allows them to reset and re-think the research plan to come up with more successful ideas (Harrick et al., 1986; De Bloom et al., 2014).

In the long-term, after experiencing these challenges, researchers may be more flexible and mature when facing negative situations. Negative situations include another global pandemic, wars, social conflicts, bias and discriminations, negative research results, and any other situations that may bring stress (Zarei et al., 2014).

## Strengths and Weaknesses of the Study

Our study is unique in that it is designed to maintain well-being and improve the productivity of basic science researchers during the COVID-19 era. Although it is a small case study with only 19 participants (a limitation), the study provides preliminary evidence that the strategy has a positive impact on participants’ well-being and productivity. Moreover, the study design using both cross-sectional and longitudinal studies, provides rigor to our analysis and conclusion. This study does not perform cross-sectional findings for productivity, as comparing data from other basic science research groups may be unfair and difficult. Our study may be utilized, upon optimization, by a specific group to manage the well-being of their research group and maintain productivity during a challenging situation like COVID-19.

## Implications and Future Prospects

From the corporate perspective, all industries have been affected during COVID-19 pandemic, including the energy, tourism, transportation, and retail and manufacturing sectors (Fu and Shen, 2020; Shen et al., 2020). For instance, the performance of companies belonging to energy sectors is found to be negatively impacted in a study performed on the corporate performance in the energy industry by the panel data and Difference-in-Difference model (Fu and Shen, 2020). Therefore, this study could be implemented with or without modifications in every sector to improve the well-being of individuals and enhance their productivity. More specifically, the strategies discussed in this study could be highly beneficial when implemented in healthcare and higher education institutions.

As a vaccine for COVID-19 has not yet been approved, and due to the resurgence of the infection a future limited lock-down may yet take place. Therefore, it is important to continue to optimize the current approach if similar circumstances recur. Due to current fears for a second wave of illness during the flu season, which may further complicate the diagnosis and treatment of COVID-19, it will be beneficial to continue to monitor PSS and COVID-SS regularly. Thus, this finding will provide a potential measure for other research groups to take necessary steps in managing well-being and maintaining productivity in case the second wave leads to either full or partial lock-down and/or lab closures. Furthermore, the second wave of illness will necessitate extra caution in practicing preventive health measures. Research groups, as well as groups in other professions, could use similar empowerment sessions to encourage each other to keep healthy diets, meet exercise goals, and maintain regular sleep schedules, to the extent that their occupations allow.

Finally, the strategy discussed in this study, upon appropriate modification to tailor the situation, could also be implemented in other future challenges that we may face, e.g., new emerging or re-emerging epidemics or pandemics, financial crises, natural disasters, etc. Based on historical perspectives, either locally or globally, we face financial crises and epidemics every decade, as well as natural disasters in multiple countries almost every year (Archer and Geyer, 1982; Roser, 2019; Financial Times, 2020). Therefore, it is important to have a strategy at every institution, especially at research and educational institutions, to effectively mitigate the stress and anxiety caused by these challenges and to improve the well-being and productivity of individuals.

## Data Availability Statement

The raw data supporting the conclusions of this article will be made available by the authors, without undue reservation, to any qualified researcher.

## Ethics Statement

The studies involving human participants were reviewed and approved by the Institutional Review Board, University of Tennessee Health Science Center. The patients/participants provided their written informed consent to participate in this study.

## Author Contributions

SaK conceived of the presented idea, obtained and analyzed the data, and wrote the first draft of the manuscript. SuK obtained and analyzed the data, and wrote part of the manuscript. AK obtained data, and wrote part of the manuscript. KG obtained data, and wrote part of the manuscript. KZ Obtained additional data and contributed significantly for revision of the manuscript.

## Conflict of Interest

The authors declare that the research was conducted in the absence of any commercial or financial relationships that could be construed as a potential conflict of interest.
